# Evaluating early EEG correlates of restricted and repetitive behaviors for toddlers with or without autism

**DOI:** 10.21203/rs.3.rs-3871138/v1

**Published:** 2024-01-18

**Authors:** Haerin Chung, Carol L. Wilkinson, Alex Job Said, Helen Tager-Flusberg, Charles A. Nelson

**Affiliations:** Boston Children’s Hospital; Boston Children’s Hospital; Boston Children’s Hospital; Boston University; Boston Children’s Hospital

## Abstract

**Background:**

Restricted and repetitive behaviors (RRB) are among the primary characteristics of autism spectrum disorder (ASD). Despite the potential impact on later developmental outcomes, our understanding of the neural underpinnings of RRBs is limited. Alterations in EEG alpha activity have been observed in ASD and implicated in RRBs, however, developmental changes within the alpha band requires careful methodological considerations when studying its role in brain-behavior relationships during infancy and early childhood. Novel approaches now enable the parameterization of the power spectrum into periodic and aperiodic components. This study aimed to characterize the neural correlates of RRBs in infancy by (1) comparing infant resting-state measures (periodic alpha and aperiodic activity) between infants who develop ASD, elevated likelihood infants without ASD, and low likelihood infants without ASD, and (2) evaluate whether these infant EEG measures are associated with frequency of RRBs measured at 24 months.

**Methods:**

Baseline non-task related EEG data were collected from 12-to-14-month-old infants with and without elevated likelihood of autism (N=160), and periodic alpha activity (periodic alpha power, individual peak alpha frequency and amplitude), and aperiodic activity measures (aperiodic exponent) were calculated. Parent-reported RRBs were obtained at 24 months using the Repetitive Behavior Scale-Revised questionnaire. Group differences in EEG measures were evaluated using ANCOVA, and multiple linear regressions were conducted to assess relationships between EEG and RRB measures.

**Results:**

No group-level differences in infant EEG measures were observed. Marginal effects analysis of linear regressions revealed significant associations within the ASD group, such that higher periodic alpha power, lower peak alpha frequency, and lower aperiodic exponent, were associated with elevated RRBs at 24 months. No significant associations were observed for non-ASD outcome groups.

**Limitations:**

The sample size for ASD (N=19) was modest for examining brain-behavior relations. Larger sample sizes are needed to increase statistical power.

**Conclusion:**

For infants with later ASD diagnoses, measures of alpha and aperiodic activity measured at 1-year of age were associated with later manifestation of RRBs at 2-years. Longitudinal studies are needed to elucidate whether the early trajectory of these EEG measures and their dynamic relations in development influence manifestations of RRBs in ASD.

## Introduction

Restricted and repetitive behaviors (RRBs) are core features of autism spectrum disorder (ASD). Examples of RRBs include motor movements (e.g. hand-flapping or arm-waving), as well as complex cognitive behaviors like intense preoccupation with specific objects (e.g. peering at the parts of toys) or topics (e.g. having specific knowledge about trains). While many RRBs are not unique to infants later diagnosed with ASD early in infancy [1], studies indicate that developmental trajectories of these behaviors diverge from typically developing infants. For instance, common repetitive actions such as object banging are not uncommon in typical infant development but decrease with age, whereas various types of RRBs remain elevated in children with ASD (RRBs measured via diagnostic observation [2, 3] and parent questionnaires [4, 5]). Some types of RRBs, such as “stimming”, have been reported to help regulate sensory inputs in autistic adults and should not be universally considered “negative” [6]. However, there is also evidence that the severity of certain RRBs is associated with worse developmental outcomes. As an example, during early development, time spent engaging in RRBs may negatively impact learning opportunities, limiting the scope for exploration and varied experiences [7]. Additionally, higher levels of RRBs (preoccupations with parts of objects, sensory interests, and stereotyped motor movements) at 3–5 years predict lower cognitive and adaptive skills, along with an increased prevalence of autistic behaviors measured via the Childhood Autism Rating Scale (CARS; [8]) at 8–10 years [9]. Thus far, limited research has focused on understanding the neural underpinnings of RRBs in autism with only a handful of neuroimaging studies of infancy identifying differences in brain activity related to RRBs [10–12]. Improved characterization of early RRB development holds potential for earlier detection of ASD and increasing our understanding of how the presence or persistence of various RRBs impact long-term developmental outcomes.

Electroencephalography (EEG) is commonly employed in autism research to investigate differences in brain activity and development. Alpha oscillations, predominantly observed in the parietal-occipital regions (adults: 8–12 Hz; infants/toddlers: 6–9Hz; [13]), play a pivotal role in sensory perception (e.g., somatosensory: [14]; auditory: [15], top-down cognitive control processes [16, 17], inhibitory functions and selective attention [18, 19]). Thus, neural activity in the alpha frequency is a promising neural correlate of RRBs in ASD, however only a handful of studies have investigated EEG measures in the alpha range associated with RRBs. For instance, resting-state parietal alpha power in adults was found to be positively associated with behavioral rigidness using the Broad Autism Phenotype Questionnaire (BAP-Q) [20]. In infants, Orekhova et al., (2014) explored alpha connectivity in relation to RRBs in 14-month-olds with and without later diagnosis of autism. Significant group-level differences in global alpha connectivity were found along with a significant association between early hyper-connectivity in frontal-to-other-regions and future severity of RRBs in toddlers with ASD [11]. This relationship between increased alpha connectivity and elevated RRBs in toddlerhood, but not group-level differences in alpha connectivity, was replicated, providing further support for the connection between alpha activity and the manifestation of RRBs in ASD during early development [12].

Alpha power measured during non-task related baseline has also been studied more generally in ASD; however, findings are often conflicting. Some studies report decreased alpha power [21, 22] while others find no differences [23, 24] between autistic children compared to typically developing counterparts (see for a review). Mixed findings may be related to the complexity of developmental changes that occur within alpha during early development. For example, the frequency at which oscillations in the alpha range demonstrate maximal power (i.e., peak alpha frequency - PAF), increases with age [25–27]. As alpha continues to develop from infancy throughout childhood, calculated alpha power is impacted by the frequency range that is selected. Using a fixed frequency band that does not accommodate developmental changes in peak frequency may contribute to mixed findings. Relatedly, differences in the rate of change in PAF may also exist in neurodevelopmental disorders; while PAF tracks with chronological age in neuro-typical development [25–27], in ASD, PAF is associated with non-verbal cognitive metrics, rather than chronological age [28]. Interestingly, while Orekhova et al. (2014) explored both alpha power and alpha connectivity, significant associations with RRBs in ASD were only observed for alpha connectivity. The lack of association between alpha power and ASD may have been influenced by using a narrow-fixed frequency band (7–8Hz), potentially obscuring individual differences both between and within groups. Thus, it is important to account for developmental variation in alpha peak and power when assessing alpha activity early in development in neurotypical and neurodiverse populations.

Given concerns about power measures, novel approaches have been developed to decompose aperiodic and periodic components of the EEG power spectrum, avoiding the constraints associated with predefined canonical frequency bands of interest [29, 30]. The aperiodic component, reflecting broadband, non-oscillatory, neural activity is defined by an exponential decrease in power across increasing frequencies that follows approximately a 1/f distribution, with the aperiodic exponent defined as χ in the 1/fχ formulation of the spectrum. The periodic component reflects oscillatory activity manifesting as peaks in the spectrum at specific frequencies, where power rises above the aperiodic curve [31]. Separating out periodic and aperiodic components enhances precise estimation of periodic activity, including measures of peak alpha amplitude (PAA) and PAF [32, 33].

With this in mind, studies have begun to clarify the role of periodic power and aperiodic activity measures and their relationship with cognition and behavior across the lifespan in both clinical and non-clinical populations. For instance, Cellier et al., (2019) parameterized EEG data from 3–24 years and observed decreases in aperiodic exponent with age, as well as age-dependent increases in peak frequency from 4 to 12 Hz measured over posterior midline (POz). Moreover, the aperiodic exponent holds promise for mechanistic explanations in neurodevelopmental disorders. Particularly, the aperiodic exponent, or 1/f slope, is thought to reflect the balance between excitation (E) and inhibition (I) (i.e. E/I) [35], with a steeper slope (higher aperiodic exponent) indicating greater inhibition relative to excitation. Studies have begun to explore whether differences in the aperiodic exponent exists in clinical conditions where imbalance in E/I has been implicated. This includes ASD [36], as well as attention deficit/hyperactivity disorder [37, 38], neurofibromatosis type 1(NF1) [39], schizophrenia; [40], and Fragile X Syndrome [41]. While interpreting the aperiodic exponent is inherently complex due to effects of developmental compensation for early alterations in E/I balance, promising insights are emerging. Notably, in NF1, a genetic condition characterized by alterations in E/I balance with preferential NF1 gene expression in inhibitory neurons, Carter Leno et al., (2022) observed higher aperiodic exponents, indicative of greater inhibition relative to excitation, in 10-month-old infants with versus without NF1. This adds support to the role of aperiodic exponent in delineating E/I balance early in infancy. Whether the aperiodic exponent is predictive of RRBs later in development has yet to be explored.

### Current Study

In the current study, we investigated the relationship between infant posterior alpha and aperiodic activity and later RRB frequency in infants with and without a later autism diagnosis. Infants were recruited from three likelihood groups: (1) infants with social communication delays based on parent survey at 12 months (Screener group), (2) infants with an older sibling with ASD (Sib group), and (3) infants without an older sibling with ASD without social communication delays at 12 months (Low Likelihood group). Infants were longitudinally followed with autism outcomes determined at 24 and 36 months. First, we tested whether parameterized alpha and aperiodic activity EEG measures at 12–14 months differ between infants with and without a future ASD diagnosis. Second, we tested whether these EEG measures at 12–14 months were associated with frequency of RRBs at 24 months.

## Method

### Participants

Participants were recruited as part of the Infant Screening Project, a longitudinal study conducted in collaboration with Boston Children’s Hospital/Harvard Medical School and Boston University. All participants were born with minimal gestational age of 36 weeks, birth weight over 2.5kg, and with no known genetic or neurological disorders. This was a prospective longitudinal study with infants recruited based on three different likelihoods of receiving a later ASD diagnosis: (1) Elevated familial likelihood infants had an older sibling with a confirmed ASD diagnosis (Sib); (2) Elevated Screening likelihood infants were defined by low scores on the Communication and Symbolic Behavior Scales (CSBS) parent-questionnaire at 12–14 months of age (Screener) [42]. Low CSBS scores were considered as those at or below 1.25 standard deviations below the mean, with cutoff scores of 27 for 12 months, 28 for 13 months, and 32 for 14 months out of a total score of 57; (3) Low Likelihood (LL) participants consisted of infants who had no immediate family history of ASD and passed the CSBS screener.

Autism outcomes were conducted at 24 and 36 months of age, however a number of participants reached 24 or 36 months during the COVID pandemic when in-person visits were not possible. In-person evaluations included the Autism Diagnostic Observation Schedule-Second Edition (ADOS-2; [43]), the Mullen Scales of Early Learning (MSEL; [44]), and a parent-child interaction. During COVID pandemic, remote evaluations included the Brief Observation of Symptoms of Autism (BOSA; [45]), parent-child interaction, the Autism Symptom Interview (ASI; [46]), and the Vineland Adaptive Behavior Scales – Third Edition, Parent Interview Form (Vineland-3; [47]). For toddlers meeting criteria on the ADOS/BOSA or coming within three points of cutoffs, a licensed clinical psychologist reviewed assessment scores and videos and provided their best clinical judgment regarding an ASD diagnosis. 140 participants were assessed via the ADOS and 20 were assessed via the BOSA. Final outcome groups were defined by ASD diagnosis and likelihood group, resulting in the following four groups: (1) ASD (2) Sib-noASD, (3) Screener-noASD, and (4) LL-noASD. Sample characteristics across groups are shown in Table 1. The analysis included a total of 160 participants at 12–14 months.

### Behavioral measures and questionnaires

#### Communication and Symbolic Behavior Scales, Developmental Profile (CSBS)

The CSBS [42] is a standardized assessment scale designed for screening for developmental delays and evaluating social communication and language skills for infants and toddlers between 6 and 24 months of age. The CSBS includes 24 questions and assesses both language skills and symbolic development, including gestures, facial expressions, and play. Questions are rated on a 3-point scale, with the following categories: Not yet, sometimes, and often.

#### Autism Diagnostic Observation Schedule-Second Edition (ADOS-2)

The ADOS-2 [43] is a semi-structured standardized observational assessment designed to evaluate the current level of ASD symptoms of the participant. During this session, the responses and behaviors of toddlers are systematically observed and rated on two primary components: (1) Social affect, which assesses aspects of social communication, interaction, play/imagination and (2) Restricted and repetitive behavior, focusing on the presence and severity of behaviors indicative of restricted interests and repetitive actions.

#### Mullen Scales of Early Learning (MSEL)

The MSEL [44] is a standardized developmental assessment for children 0–68 months of age. The non-verbal and verbal developmental quotients are calculated based on the age equivalents of the 4 subscales (non-verbal: Fine Motor, Visual Reception; verbal: Expressive Language, and Receptive Language) and the child’s chronological age.

#### Brief Observation of Symptoms of Autism (BOSA)

The BOSA [45] is an assessment for autism which consists of a 12–14 min interaction between an individual and a caregiver or clinician, which can be administered in both in-person and telehealth settings. The BOSA offers a social context with standardized materials and activities, adapted from the Brief Observation of Social Communication Change [48] and ADOS-2, and can be coded and evaluated by clinicians trained in the ADOS. High convergent validity between BOSA and ADOS-2 were established (Toddler Module Overall Total and Calibrated Severity Score: 0.74 (p < 0.001); [45]).

#### Autism Symptom Interview (ASI)

The ASI [46] is a brief (15–20 min) caregiver interview designed to facilitate quick classification of children with ASD for research studies. With items derived from the Autism Diagnostic Interview-Revised [49], the ASI includes 55 questions in 4 domains of functioning: language, social communication, peer interaction, and restricted and repetitive behaviors, as well as diagnostic history of the child.

#### Vineland Adaptive Behavior Scales– Third Edition, Parent Interview Form (Vineland-3)

The Vineland-3 [47] is a semi-structured interview with a caregiver which assesses a child’s day-to-day adaptive functioning across four domains including communication, daily living skills, socialization, and motor skills. Scores for each item range from 0 to 2 with the following categories: the participant never performs this action (score = 0), the participant sometimes or is partly capable of performing this action (score = 1), or the participant performs the action in daily life (score = 2).

#### Repetitive Behavior Scale-Revised (RBS-R)

The RBS-R [50] is a 44-question survey designed to evaluate RRBs in individuals with autism across development. It covers six subdomains of RRBs: stereotyped, self-injurious, compulsive, routine, sameness, and restricted behaviors. Caregivers rate each behavior on a 4-point scale, indicating the frequency of occurrence of each behavior in the past month. Scale ranges from 0 to 3, with the following categories: behavior does not occur (score = 0), behavior occurs and is a mild problem (score = 1), behavior occurs and is a moderate problem (score = 2), behavior occurs and is a severe problem (score = 3).

### EEG Assessment

#### EEG Data Acquisition

Two-to-five minutes of non-task-related baseline EEG data were acquired while infants seated on their caregiver’s lap, watched a video of abstract moving shapes in a dimly lit, sound-attenuated room. Research assistants refrained from social interaction with the infant but sometimes blew bubbles across the room or presented a quiet toy (e.g., a ball) to the infant if they became fussy. EEG data were collected with 128 channel Geodesic Sensor Nets connected to a NetAmps 300 amplifier, sampled at 500Hz with a 0.1 Hz high-pass analog filter, and online re-referencing to the vertex (Cz) through NetStation software (Electrical Geodesics, Inc (EGI), Eugene, OR, USA). Impedances were kept below 100KΩ in accordance with the impedance capabilities of the high-impedance amplifiers inside an electrically shielded room.

#### EEG pre-processing

**Figure 1:**
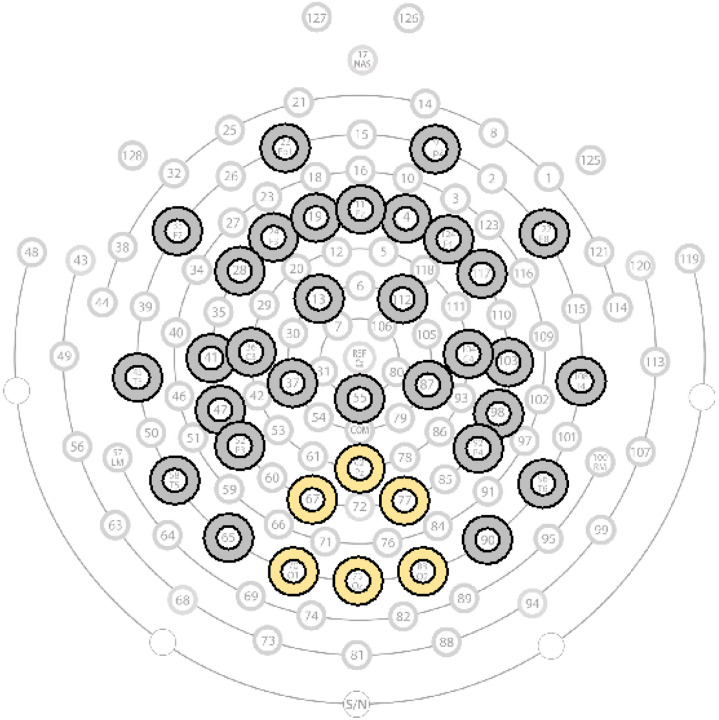
Electrode layout: 128-channel Hydrocel Geodesic Sensor Net. Grey and yellow circles denote electrodes included in ICA and MARA steps of pre-processing. Yellow circles indicate electrodes averaged for posterior regions of interest.

The continuous EEG data were first exported from NetStation to MATLAB format (version R2017a). Data preprocessing, artifact removal, and data quality assessment were carried out via the Harvard Automated Processing Pipeline for EEG (HAPPE 1.0; [51], a preprocessing pipeline optimized for developmental EEG data). All files were batch processed using the Batch Electroencephalography Automated Processing Platform (BEAPP; [52]) software. In order to optimize artifact rejection performance given the short lengths of EEG in infant data, a spatially distributed subset of channels providing whole-head coverage was processed through HAPPE (electrodes: 9, 11, 22, 24, 33, 124, 122, 36, 45, 52, 58, 62, 70, 83, 92 ,96, 104, 108, 122, 124, 28, 19, 4, 117, 13, 112, 41, 47, 37, 55, 87, 103, 23, 98, 65, 67, 77, 90, 75; Fig. 1). For each EEG, a 1 Hz digital high-pass filter and a 100 Hz low-pass filter was applied in preparation for independent component analysis (i.e. ICA). Data were then resampled with interpolation to 250 Hz (resampling was performed after filtering to avoid aliasing higher frequencies when resampling). Electrical line noise was removed at 60 Hz via CleanLine [53]. Artifacts were rejected (e.g., eye blinks, movement, and muscle activity) through wavelet-enhanced ICA with automated component rejection via EEGLAB [54] and the Multiple Artifact Rejection Algorithm [55, 56]. Post-artifact rejection, any channels removed during the bad channel rejection were interpolated through spherical interpolation to reduce spatial bias in re-referencing. The EEG data were then re-referenced to the average reference, detrended to the signal mean, and segmented into contiguous 2-s windows. Additional segments were then rejected using HAPPE’s amplitude and joint probability criteria.

#### EEG rejection criteria

EEG recordings were rejected using the following HAPPE data quality measures: Fewer than 20 segments (40 seconds of total EEG), percent good channels < 80%, percent independent components rejected > 80%, mean artifact probability of components kept > 30%, and percent variance retained < 25%. All data quality metrics were similar across outcome groups (Table 2). Of 178 participants with EEG recordings, 13 were excluded due to fewer than 20 segments, and 5 were excluded for having more than 80% good channels.

#### EEG Spectral decomposition and parameterization analysis

The power spectral density at each electrode, for each 2-second segment, was calculated in the BEAPP Power Spectral Density (PSD) module using a multitaper spectral analysis with three orthogonal tapers. For each electrode, the PSD was averaged across segments, and then further averaged across posterior regions of interest (electrodes: 70, 75, 83, 67, 77, 62; Fig. 1). This posterior electrode cluster over the parietal-occipital cortex was determined *a priori* and was selected based on 1) prior research indicating that the dominant oscillation of alpha typically originates in parietal-occipital cortex [13, 34] and 2) Carter-Leno et al., (2018)’s finding on the relation between parietal alpha power and BAP-Q.

The PSD was then further analyzed using a modified version of SpecParam v1.0.0 (Also known as FOOOF, https://github.com/fooof-tools/fooof; in Python v3.6.8) to model periodic and aperiodic components of the power spectra. SpecParam required modification for use in this age range, as power spectrum models showed poor model fit (increased mean squared error) for frequencies between 10–20Hz (see Wilkinson et al., 2023 for more details on modifications to the original algorithm). The SpecParam model was used in the fixed mode (no spectral knee) with peak_width_limits set to [0.5, 18.0], max_n_peaks = 7, and peak_threshold = 2. Further analyses were subsequently restricted to 2.5–50Hz given elevated error between 2–2.5, and 50–55Hz. Mean *R*^2^ for the full sample was 0.997 (STD = 0.009). Mean estimated error for the sample was 0.01 (STD = 0.010). The mean *R*^2^ and mean estimated error for each group was as follows: Sib-noASD (*R*^2^ = 0.997, STD = 0.005; Error = 0.012, STD = 0.008), Screener-noASD (*R*^2^ = 0.998, STD = 0.012; Error = 0.013, STD = 0.008), LL-noASD (*R*^2^ = 0.996, STD = 0.012; Error = 0.014, STD = 0.011), and ASD (*R*^2^ = 0.997, STD = 0.005; Error = 0.012, STD = 0.010).

**Figure 2:**
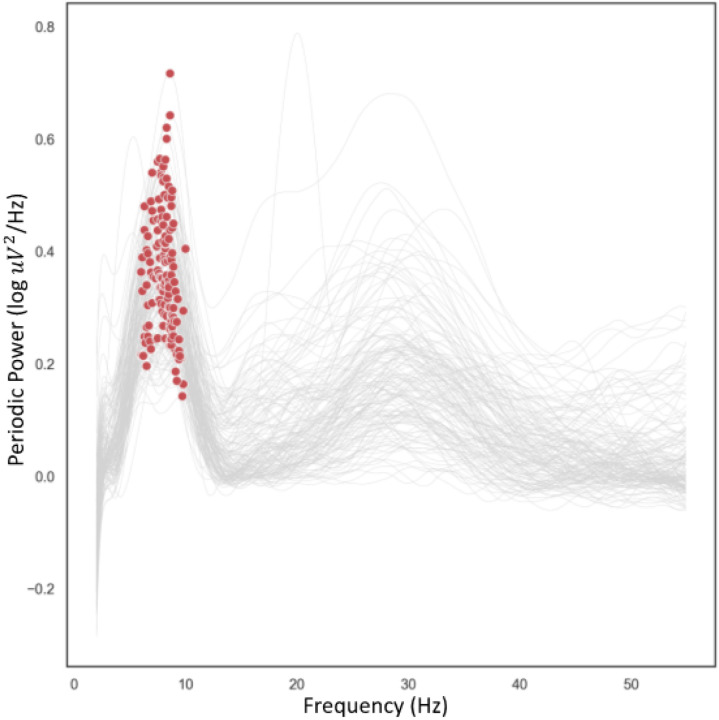
Periodic power spectra indicating peak frequency. 12–14 month individual periodic power spectral density (PSD) at 2–50 Hz for all participants at posterior region of interest. Grey lines indicate individual participants’ power spectra. Red dots indicate the maxima in which peak alpha frequency and amplitude were identified. Power values are log transformed.

SpecParam outputs include an aperiodic offset and slope to describe the estimated aperiodic 1/f signal. Each individual’s periodic power spectrum was determined by subtracting their SpecParam-estimated aperiodic spectrum from their original absolute power spectrum. Spectral analysis was performed to get a measure of periodic alpha power by binning power of each detected oscillation into the following the traditional “canonical” alpha band: 6–9 Hz. To characterize individual PAA and PAF, the periodic spectrum was smoothed using a savgol filter (scipy.signal.savgoal_filter, window length = 101, polyorder = 8). Maxima were identified within 6 − 12 Hz, and the frequency and amplitude for the maxima were extracted. Only those with an identifiable peak in the 6 − 12 Hz range was included in the analyses for PAA and PAF. We selected this frequency range based on previous developmental work suggesting 6 Hz is an appropriate lower bound for the alpha rhythm in infants from 5 months upwards [58], and extended our upper bound to 12 Hz to enable identification of peaks that are outside of the canonical band. Figure 2 displays PSDs for the whole sample to illustrate there was a clear peak in the 6 − 12 Hz range. There were two participants where FOOOF identified two peaks in 6 − 12 Hz range in the posterior ROI; in these participants the higher value was taken as their PAF. Peaks were not identified in eleven participants. To characterize the aperiodic exponent (slope of the spectra), we used χ in the 1/fχ model fit.

### Statistical Analyses

All statistical analyses and visualizations were done using the Python and R programming language. To explore whether EEG at 12–14 months differed between outcome groups, we performed a one-way analysis of variance with covariates (ANCOVA) with each EEG measure (periodic alpha power, PAF, PAA, or aperiodic exponent) as the dependent variable, outcome groups as a between-participant variable, and age (in days), sex, and non-verbal MSEL as a covariates.

To evaluate the effect of outcome group on the relationship between EEG measures (periodic alpha power, PAF, PAA, or aperiodic exponent) and RRBs, linear regression models included a two-way interaction between outcome group and the relevant EEG measure (setting ASD group as the main reference group). As there were more males in the Screener-noASD and ASD group, sex was included as a covariate in all models. In addition, since our EEG measures were collected between 12-to-14 months, and as previous research found associations between age and cognitive measures with EEG measures (alpha power and PAF), age and non-verbal MSEL were included as covariates in all the linear regression models. Standardize_parameters function in the R package *effectsize* (version 0.0.1; [59]) was used to re-fit and compute standardized model parameters (standardized coefficients). To characterize interaction effects within the models, marginal effects analyses [60] were performed. R package *emmeans* [61] and *ggplot2* [62] were used to plot estimated regression lines and the interaction plot of estimated marginal effects. Post hoc comparisons were conducted between sub-groups using the Tukey method at 95% confidence level. In addition, we computed spearman correlations within each group to measure the magnitude of the relation between EEG at 12–14m and RRBs at 24m.

1. Model = RRB at 24m ~ EEG measure * Outcome group + age(days) + sex + non-verbal MSEL
2. Standardize_parameters (model, method = “refit”) for standardized coefficients
3. emmeans to conduct post hoc analyses: marginal effects of interaction of EEG and Outcome group.
4. Spearman correlations

## Results

### Participant characteristics

A total of 160 infants contributed EEG data to this analysis. Demographic data are shown in Table 1 for the full sample, as well as separated by the four outcome groups: LL-noASD, Screener-noASD, Sib-noASD, and ASD. 18.7% of participants identified as a race other than white, and 74.3% reported income levels above $100,000. Outcome groups differed in terms of sex (χ^2^ = 13.4, p = 0.004) in that the Screener-noASD and ASD groups were mostly male (around 80%). MSEL verbal and non-verbal quotients were assessed at 12–14 months. Outcome groups significantly differed on MSEL verbal quotients (F(3, 153) = 4.4, p < 0.05; see Table 1). Post-hoc Tukey HSD test indicated there were significant differences between Screener-noASD and LL-noASD (mean difference = 11.05, adj-p = 0.03) and between Screener-noASD and Sib-noASD (mean difference = 12.99, adj-p = 0.02). (Note that the verbal MSEL score for ASD (mean = 93.74) is comparable to the score for those in the Screener-noASD group (mean = 90)). There were no significant group differences for MSEL non-verbal quotients. As expected outcome groups significantly differed on RRB measured at 24 months (F(3, 129) = 13.5, p < 0.001). Post-hoc Tukey HSD test indicated there were significant differences between ASD and LL-noASD (mean difference = 10.09, adj-p < 0.001), between ASD and Sib-noASD (mean difference = 9.07, adj-p < 0.001), and between ASD and Screener -noASD (mean difference = 6.22, adj-p = 0.02).

### EEG measures at 12–14 months

**Figure 3:**
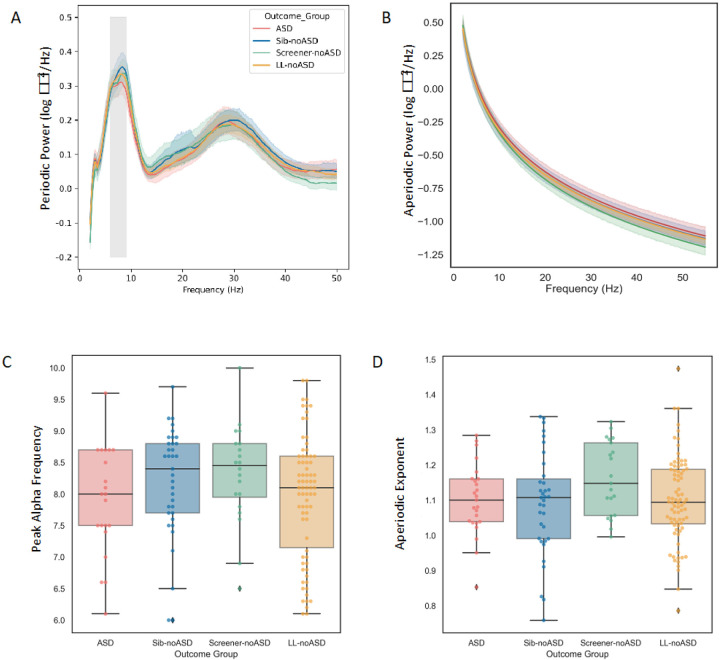
Periodic and Aperiodic Power Spectra at 12–14 months. (A-B) Periodic and Aperiodic power spectra averaged by Outcome group. (A) Canonical alpha frequency band (6–9Hz) shaded in grey. (C–D) Boxplots of Peak alpha frequency and Aperiodic exponent by Outcome group.

First, we examined group-level differences in periodic alpha power, PAA, PAF, and aperiodic exponent. All 12–14 month EEG measures were similar across outcome groups (periodic alpha power (*F*(3, 122) = 0.26, p = 0.85), PAA (*F*(3, 112) = 0.23, p = 0.87), PAF (*F*(3, 112) = 1.72, p = 0.17), and aperiodic exponent (*F*(3, 122) = 1.00, p = 0.39); see Fig. 3). Mean values of EEG measures at 12–14 months are reported in Table 1.

### EEG measures at 12–14 months and RRBs at 24 months

**Figure 4:**
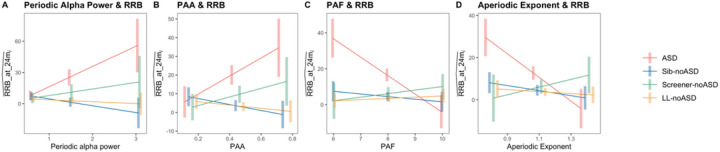
Association between EEG at 12–14 months and Estimated RRB at 24 months. (A) Periodic alpha power (B) PAA (C) PAF (D) Aperiodic exponent and estimated RRB by Outcome group. Lines represents the best fitting regression line with marginal means, and confidence intervals represent Tukey adjusted 95 % confidence intervals.

**Figure 5:**
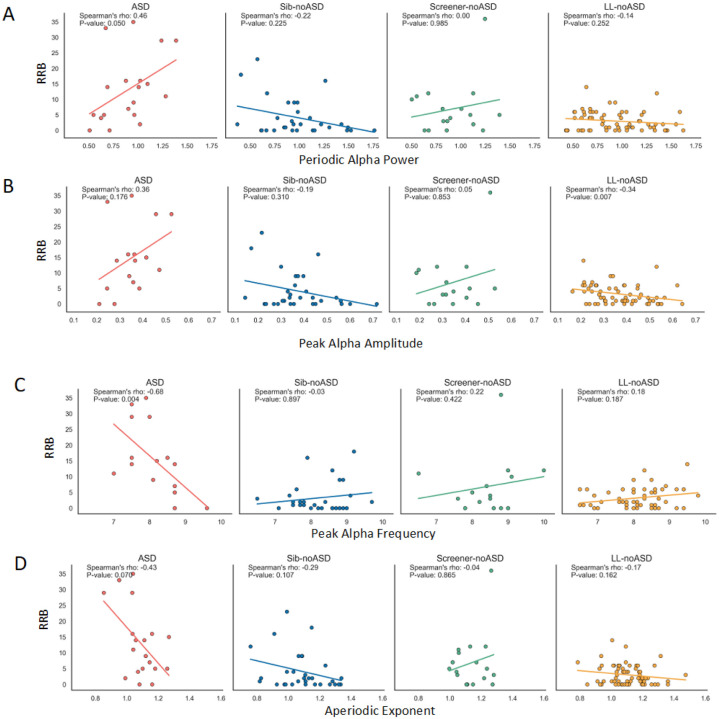
Scatterplot between EEG at 12–14months and RRBs at 24 months of (A) Periodic alpha power (B) PAA (C) PAF (D) Aperiodic exponent and RRBs for each Outcome group. Dots indicate actual values and lines represent regression lines.

Next, we assessed whether EEG measures at 12–14 months were associated with parent reported RRBs at 24 months. All four models indicated a significant interaction effect between EEG and outcome group on RRB scores, suggesting that brain-behavior relationships were different between groups. The full output for linear regression models of all four EEG measures is reported in Table 3. Overall, several significant associations, accounting for multiple comparisons, were observed:

1. Simple slopes from all four models revealed that only ASD infants showed a significant relationship between EEG and RRBs (Periodic alpha power- slope:19.49, *p* < 0.01; PAA- slope: 48.23, *p* < 0.05; PAF- slope: −10.24, *p* < 0.001; aperiodic exponent- slope: −56.15, *p* < 0.05; see Table 4). Within the ASD group, periodic alpha power and PAA showed positive associations with RRBs, while PAF and aperiodic exponent showed negative associations with RRBs. Estimated marginal means of the interaction between EEG measures and outcome groups are visualized in Fig. 4 with 95% confidence intervals. Correlation plots of each EEG measure and RRBs are visualized in Fig. 5 for each outcome group.
2. The significance of the interaction terms represents whether the slope of the evaluated association is significantly different between ASD (the reference group) and the three likelihood groups without ASD. The slopes of EEG measures versus RRB scores across all models for ASD infants were significantly different from both Sib-noASD and LL-noASD infants. Significant slope differences between ASD and ScreenernoASD infants were observed for PAF and aperiodic exponent (but not alpha power or PAA) models (see Table 3 for contrasts between slopes). We note that within the Screener-noASD group there is an outlier with high RRB score that may be driving relationships within this small sample size (Fig. 5).

Together, our findings indicate that for only ASD infants, higher periodic alpha power, higher PAA, lower PAF, and lower aperiodic exponent (indicative of shift towards E > I) at 12–14 months, were associated with elevated RRBs at 24 months.

## Discussion

In the current study we sought to explore whether early resting-state EEG measures were associated with later frequency of RRBs in infants with and without elevated likelihood of ASD who do or do not go on to receive an ASD diagnosis. There were no outcome group-level differences in 12–14 month EEG measures (periodic alpha power, PAF, PAA, and exponent). However, specific to the ASD group, we observed notable associations between 12–14 month EEG resting-state measures and RRBs reported at 24-months, with increased periodic alpha power, and decreased peak alpha frequency and aperiodic exponents, associated with elevated RRBs.

Prior literature investigating alpha power measures in ASD have been mixed. Consistent with our findings, four other studies (three using parameterized EEG components [63–65]) have also reported no group-level differences between ASD and typically developing counterparts. Two studies of older children (6 years and older) have reported no group differences in alpha power and PAF [23], and no group differences in alpha amplitude and other features of waveforms in the alpha-band (i.e. characterizing asymmetry of waveforms by cycle) [65]. Similarly, no differences in PAF were observed in studies involving infants with TSC [64] and infants with autistic siblings [63], with and without later diagnosis of ASD. However other studies in older children have observed differences in alpha power and PAF in children with and without ASD [28, 66, 67]. Mixed results may stem from methodological variations, such as differences in exploring alpha power with and without spectral parameterization, along with the diversity of age ranges in cross-sectional studies.

Given hypotheses that ASD is characterized by an imbalance in excitation and inhibition [68], and prior research suggesting that the aperiodic exponent reflects E/I balance [35], we explored whether ASD infants would have decreased aperiodic exponent (increased E/I) compared to other outcome groups. However, no group-level differences were observed. Similarly, Cater-Leno et al., (2022) found that 10-month infants with and without autistic siblings have similar aperiodic exponents, and Plueckebaum et al., (2023) found that children (7–17 years) with and without ASD have similar E/I measure.

It is important to note that our analysis is cross-sectional and unable to account for group differences in EEG developmental trajectories. While the lack of group-level distinctions in EEG metrics at 12–14 months could be a genuine observation, it signals the need for a more comprehensive investigation into longitudinal changes in alpha oscillations or aperiodic activity across ASD and other likelihood groups across early childhood. Indeed, studies spanning across multiple ages have observed notable differences in the *trajectories* of EEG measures between ASD and typically developing children [69–71]. As such, more comprehensive investigation of longitudinal changes in both periodic and aperiodic activity, is essential to capture variations over time that may elucidate the functional relationships between EEG and RRBs in ASD.

We replicated previous findings in adults that higher alpha power is associated with more RRBs in ASD. Expanding upon existing literature, we also observed that lower infant PAF is associated with increased RRBs at 24 months. Why might alpha activity in infancy impact later RRBs? Alpha-band activity has been postulated to play a crucial role in maintaining an active and adaptive inhibitory mechanism during perceptual processing, with studies finding alpha to be associated with gating and suppressing irrelevant information, as well as selective attention and engagement [19]. One possible explanation is that heightened alpha power may reflect increased internal focus, excessive inhibition, or suppression of information, potentially resulting in heightened attention and reduced flexibility in adapting to diverse contexts [72].

In pediatric populations, PAF increases with age from infancy to childhood [26] and developmental increases in PAF have been linked to maturation of brain connections, facilitating efficient neural communication and in turn, supporting increasing cognitive competence [13, 59]. Here we hypothesize that lower PAF in ASD may reflect a more immature brain infrastructure with less efficient neural networks, leading to prolonged maintenance of RRBs later in toddlerhood. Future studies with larger samples should confirm the observed positive association between higher alpha power and RRBs, as well as the negative association between lower PAF and RRBs.

While we did not observe differences in aperiodic activity between ASD outcome groups, we did observe a significant association between lower aperiodic exponent and increased RRBs. A growing body of research suggests that the aperiodic exponent reflects the balance between excitation and inhibition, with a lower (flatter) exponent reflecting high excitation over inhibition (higher E/I ratio) [35]. Our findings therefore suggest that within ASD toddlers, an increased E/I ratio is associated with higher levels of RRBs. This aligns well with the hypothesis implicating an imbalance in the excitatory (E) glutamate and inhibitory (I) gamma-aminobutyric acid (GABA) systems in the etiology of ASD [68]. For instance, a reduction of GABAergic inhibition could lead to enhanced neural noise, which may result in reduced gating [73] and less efficient information transmission in networks [74]. Thus, we speculate that an increased E/I ratio may reflect high neural noise, ultimately leading to increased RRBs in ASD. While few studies have investigated aperiodic exponent and RRBs, reduced aperiodic exponents have been reported in several movement disorders [75]; and specifically in Parkinson’s disease, lower aperiodic exponent is associated with worse motor symptom severity [76].

It is also plausible that aperiodic activity and periodic alpha activity may be developmentally linked together in the manifestations of RRBs in ASD. In the first year of life, late migrating interneurons travel from the ventral subregions into anterior regions of the brain [77], and neuronal GABA neurotransmission undergoes a developmental switch from excitatory to inhibitory [78, 79]. The timing of when these developmental changes occur holds significance as the GABAergic system directly facilitates the establishment of neural networks and oscillatory activity that are crucial for neural communication [80–83]. Thus, delays or alterations in inhibitory maturation may impact both E/I balance and the formation and maintenance of alpha oscillatory activity, thereby leading to increased RRBs in ASD. Exploring whether early developmental changes in aperiodic activity influence the establishment of alpha oscillations later in development, and how this, in turn, may impact RRBs in ASD could be a valuable avenue of further investigation.

### Limitations

There are several limitations to our study. First, while the sample size of our ASD group is larger than previous studies (current study: n = 25 compared to e.g. Orekhova et al., 2014: n = 10), it remains relatively modest, and our sample size for brain-behavior associations were further reduced in number (n = 19). Larger sample sizes are needed to increase statistical power and facilitate a more robust exploration of the utility of early EEG measures in predicting ASD diagnosis and characterizing ASD phenotypes. Second, non-ASD groups have limited variability in RRBs (as expected) and this likely impacted the ability to observe brain-behavior associations. Larger studies in populations with elevated RRBs without ASD (e.g. intellectual disability) could help understand whether our findings are specific to ASD.

## Conclusions

In conclusion, our findings demonstrate an association between EEG resting-state measures at 12–14 months in predicting RRBs at 24 months in infants with ASD. Our study contributes to the existing literature on the functional significance of alpha-band activity and RRBs in ASD. Furthermore, our findings underscore the potential utility of characterizing E/I balance via the aperiodic exponent in developmental populations. This adds to the growing body of literature on the relevance of these neurophysiological measures in understanding and predicting developmental outcomes, particularly in the context of RRBs in ASD.

## Figures and Tables

**Table 1:** Sample Characteristics at 12–14 months

	Groups Combined N = 160	LL-noASD N = 80	Sib-noASD N = 34	Screener-noASD N = 21	ASD N = 25	*P* value
**Age**, days (SD)						
	399.4 (24.05)	400.1 (23.0)	403.3 (29.4)	400.8 (17.7)	390.4 (22.9)	0.21
**Sex**, N (%)						
Female	67 (41.8)	42 (52.5)	16 (47.0)	4 (19.0)	5 (20.0)	0.004
**Ethnicity**, N (%)						0.2
Hispanic	7 (4.3)	3 (3.8)	1 (2.9)	0	3 (12.0)	
Non-Hispanic	152 (95.0)	76 (95.0)	33 (97.0)	21 (100.0)	22 (88.0)	
Not Answered	1 (0.7)	1 (1.2)	0	0	0	
**Race**, N (%)						0.15
White	130 (81.3)	62 (77.5)	27 (79.4)	17 (81.0)	24 (96.0)	
Asian	10 (6.2)	3 (3.8)	5 (14.3)	2 (9.5)	0	
Black/African American	3 (1.8)	2 (2.5)	1 (2.9)	0	0	
More than one race	16 (9.9)	12 (15.0)	1 (2.9)	2 (9.5)	1 (4.0)	
Not Answered	1 (0.8)	1 (1.2)	0	0	0	
**Income**, N (%)						0.99
<$40,000	7 (4.3)	1 (1.2)	0	2 (9.5)	4 (16.0)	
$40,000-$99,999	23 (14.3)	10 (12.5)	6 (17.1)	2 (9.5)	5 (20.0)	
>$100,000	119 (74.3)	68 (85.0)	21 (61.8)	15 (71.4)	15 (60.0)	
Not Answered or Don’t Know	11 (6.9)	1 (1.2)	7 (20.0)	2 (9.5)	1 (4.0)	
**MSEL at 12m**, M*(SD)*						
Verbal developmental quotient	99.33 (16.58)	101.66 (14.04)	103.45 (16.4)	90.6 (15.85)	93.74 (21.33)	0.006
Non-Verbal developmental quotient	114.83 (15.74)	115.87 (15.38)	116.31 (13.08)	110.51 (19.36)	113.18 (16.96)	0.48
**EEG at 12m**, M*(SD)*						
Periodic alpha power	0.93 (0.29)	0.93 (0.3)	0.96 (0.33)	0.92 (0.26)	0.88 (0.23)	0.85
PAA	0.36 (0.11)	0.36 (0.11)	0.37 (0.13)	0.35 (0.10)	0.34 (0.08)	0.87
PAF	8.04 (0.93)	7.92 (0.99)	8.21 (0.88)	8.32 (0.79)	7.88 (0.86)	0.17
Aperiodic exponent	1.11 (0.12)	1.10 (0.12)	1.09 (0.15)	1.16 (0.10)	1.10 (0.10)	0.39
**RRB Data at 24m Included in Analysis**, N												
	133	67	30	17	19	
**RRB at 24m**, M*(SD)*						
	5.18 (7.12)	3.0 (3.1)	4.03 (6.08)	6.88 (8.61)	13.11 (11.07)	< 0.001

*P* values indicate the obtained p-values from each statistical test conducted for group-level differences: ANOVA- Age, MSEL, EEG, and RRB; Chi-square test- Sex, Ethnicity, Race, and Income

**Table 2:** EEG data quality metrics

	Groups Combined N = 160	LL-noASD N = 80	Sib-noASD N = 34	Screener-noASD N = 21	ASD N = 25
**EEG quality metrics**, Mean (SD)					
Number of Segments retained after processing	97.28 (35.1)	96.91 (37.96)	102.32 (27.21)	98.05 (37.13)	90.92 (34.25)
Percent Good Channels	93 (4.2)	93 (4.2)	93 (4.5)	94 (2.6)	92 (4.6)
Percent ICs Rejected	39 (11)	40 (10)	41 (11)	35 (11)	39 (11)
Percent Variance Kept Post Waveleted Data	65.2 (14.94)	65.62 (15.57)	63.34 (14.84)	64.73 (14.16)	66.65 (14.25)
Mean Artifact Probability of Kept ICs	0.13 (0.05)	0.13 (0.05)	0.14 (0.05)	0.13 (0.04)	0.12 (0.04)

**Table 3:** Linear regression model of EEG measures and RRB

	*Periodic Alpha Power*		*PAA*		*PAF*		*Aperiodic Exponent*	
Variables	*Beta*	*std. Beta*	*Beta*	*std. Beta*	*Beta*	*std. Beta*	*Beta*	*std. Beta*
[Intercept]	12.84 [−37.83, 12.15]	1.15 [0.67, 1.63]	−11.15 [−39.12, 16.83]	1.29 [0.79, 1.80]	96.4 *** [54.19, 138.61]	1.43 [0.94, 1.93]	70.27 *** [34.63, 105.90]	0.89 [0.42, 1.36]
EEG	19.49 ** [7.74, 31.24]	0.82 [0.32, 1.31]	48.23 * [10.4, 86.06]	0.74 [0.16, 1.31]	−10.24 *** [−14.94, −5.54]	−1.29 [−1.88, −0.70]	−56.15 *** [−84.63, −27.66]	−0.99 [−1.49, −0.49]
Outcome Group								
Sib-noASD	15.27 * [2.2, 28.22]	−1.26 [−1.80, −0.73]	12.37 [−3.08, 27.83]	−1.46 [−2.02, −0.91]	−82.21 *** [−125.9, −38.56]	−1.58 [−2.12, −1.05]	−56.76 ** [−91.94, −21.58]	−1.12 [−1.64 - −0.59]
Screener-noASD	5.58 [−10.05, 21.21]	−0.94 [−1.52, −0.36]	1.01 [−16.90, 18.92]	−1.10 [−1.71, −0.50]	−108.10 *** [−159, −57.16]	−1.34 [−1.97, −0.72]	−88.21 *** [−135.9, −40.57]	−0.85 [−1.44, −0.26]
LL-noASD	9.38 [−2.44, 21.20]	−1.47 [−1.94., −0.98]	8.72 [−5.9, 23]	−1.61 [−2.12, −1.10]	−100.47 *** [−141, −59.88]	−1.71 [−2.21, −1.22]	−65.67 *** [−99.89, −31.45]	−1.22 [−1.69, −0.76]
Sex [Male]	0.22 [−2.08, 2.51]	0.03 [−0.29, 0.35]	0.40 [−2.03, 2.83]	0.05 [−0.28, 0.38]	−0.99 [−2.34, 2.32]	−0.001 [−0.32, 0.32]	0.67 [−1.57, 2.90]	0.09 [−0.22, 0.41]
Age days	0.02 [−0.02, 0.07]	0.08 [−0.08, 0.24]	0.03 [−0.02, 0.08]	0.08 [−0.08, 0.24]	0.00 [−0.04, 0.05]	0.02 [−0.14, 0.18]	0.02 [−0.03, 0.06]	0.05 [−0.10, 0.20]
Non-verbal MSEL	−0.01 [−0.09, 0.06]	−0.03 [−0.19, 0.13]	−0.01 [−0.09, 0.07]	−0.03 [−0.20, 0.14]	−0.01 [−0.08, 0.07]	−0.01 [−0.17, 0.15]	−0.02 [−0.10, 0.05]	−0.05 [−0.21, 0.11]
EEG * Outcome Group								
EEG × Sib-noASD	−26.06 *** [−39.86, −12.27]	−1.09 [−1.67, −0.51]	−64.10 ** [−106.7, −21.52]	−0.98 [−1.63, −0.33]	8.77 ** [3.46, 14.09]	1.10 [0.43, 1.77]	44.16 ** [12.01, 76.31]	0.78 [0.21, 1.34]
EEG × Screener-noASD	−13.21 [−29.80, 3.38]	−0.55 [−1.25, 0.14]	−25.32 [−74.95, 24.31]	−0.39 [−1.14, 0.37]	12.21 *** [6.07, 18.36]	1.54 [0.76, 2.31]	74.34 *** [31.87, 116.81]	1.31 [0.56, 2.05]
EEG × LL-noASD	−21.29 ** [−34.05, −8.53]	−0.89 [−1.43, −0.36]	−56.96 ** [−97.57, −16.35]	−0.87 [−1.49, −0.25]	10.93 *** [5.97, 15.89]	1.37 [0.75, 2.00]	51.53 ** [20.47, 82.59]	0.90 [0.36, 1.45]
Observations	118		108		108		118	
R^2^ / R^2^ adjusted	0.32 / 0.27	0.35 / 0.29	0.39 / 0.34	0.34 / 0.29			

* p < 0.05;

** p < 0.01;

*** p < 0.001

**Table 4:** Marginal effects of EEG measures and RRBs

	*Periodic Alpha Power*		*PAA*		*PAF*		*Aperiodic Exponent*	
Outcome Groups	δy/δx	p value	δy/δx	p value	δy/δx	p value	δy/δx	p value
Simple slopes								
ASD	19.49	0.002***	48.23	0.013*	−10.55	< .001***	−56.15	< 0.001***
Sib-noASD	−6.58	0.064	−15.88	0.093	−1.47	0.248	−11.99	0.125
Screener-noASD	6.28	0.286	22.91	0.149	1.97	0.323	18.19	0.252
LL-noASD	−1.81	0.479	−8.73	0.242	0.69	0.397	−4.62	0.445

* p < 0.05;

** p < 0.01;

*** p < 0.001

## Data Availability

Data will be available upon reasonable request.

## Abbreviations

RRB: restricted and repetitive behaviors
ASD: autism spectrum disorder
EEG: Electroencephalography
BAP-Q: Broad Autism Phenotype Questionnaire
NF1: neurofibromatosis type 1
CSBS: Communication and Symbolic Behavior Scales
ADOS: Autism Diagnostic Observation Schedule
RBS-R: Repetitive Behavior Scale-Revised
MSEL: Mullen Scales of Early Learning
BOSA: Brief Observation of Symptoms of Autism
ASI: Autism Screening Interview
PAF: peak alpha frequency
PAA: peak alpha amplitude
E/I balance: excitatory and inhibitory balance
HAPPE: Harvard Automated Processing Pipeline for EEG
BEAPP: Batch Electroencephalography Automated Processing Platform
